# Failure of cefoxitin and doxycycline to eradicate endometrial *Mycoplasma genitalium* and the consequence for clinical cure of pelvic inflammatory disease

**DOI:** 10.1136/sti.2008.030486

**Published:** 2008-04-29

**Authors:** C L Haggerty, P A Totten, S G Astete, S Lee, S L Hoferka, S F Kelsey, R B Ness

**Affiliations:** 1 University of Pittsburgh, Pittsburgh, USA; 2 Department of Medicine, Division of Infectious Diseases, University of Washington, Seattle, USA

## Abstract

**Objectives:**

As *Mycoplasma genitalium* is associated with pelvic inflammatory disease (PID), we examined the efficacy of a commonly used PID antimicrobial in treating *M genitalium* upper genital tract infection.

**Methods:**

In the PID Evaluation and Clinical Health study of inpatient versus outpatient treatment, 682 women treated with cefoxitin and doxycycline for clinically suspected PID had stored cervical and endometrial specimens available for analysis. In the current sub study, we compared baseline endometritis, short term treatment failure (continued endometritis and pelvic pain 30 days following treatment) and sequelae among women with and without *M genitalium*, identified using PCR.

**Results:**

Endometrial *M genitalium* was associated with baseline endometritis (adjusted OR 3.0, 95% CI 1.5 to 6.1). Among women with a positive baseline *M genitalium* test, 41% tested positive again 30 days following treatment. Women testing positive compared to those testing negative for *M genitalium* at baseline had an increased risk of short-term treatment failure (RR 4.6, 95% CI 1.1 to 20.1). Rates of sequelae, including infertility (22%), recurrent PID (31%) and chronic pelvic pain (42%), were high among women testing positive for endometrial *M genitalium* at baseline. There was a non-significant trend towards increased infertility, chronic pelvic pain and recurrent PID, and decreased pregnancy and live birth following *M genitalium* infection.

**Conclusions:**

*M genitalium* is associated with endometritis and short-term PID treatment failure. Cefoxitin and doxycycline, a Centers for Disease Control and Prevention recommended PID treatment regimen, is ineffective for the treatment of *M genitalium* upper genital tract infection.

*Mycoplasma genitalium* was first identified in the early 1980s among men with non-gonococcal urethritis.^1^ Because this pathogen is extremely difficult to culture, only with polymerase chain reaction (PCR) technology has research into *M genitalium* pathogenicity progressed. Numerous studies have confirmed the role of *M genitalium* in drug resistant non-gonococcal urethritis,^2^ ^3^ and some have also linked *M genitalium* with cervicitis^4^ ^5^ independent of *Chlamydia trachomatis* and *Neisseria gonorrhoeae*. Cervicitis is considered a risk factor for progression to pelvic inflammatory disease (PID)—the common infection and inflammation of the female upper genital tract, which may lead to major reproductive morbidity including infertility.^6^

Although PID has polymicrobial aetiology, with *C trachomatis* and/or *N gonorrhoeae* accounting for approximately a third to a half of cases,^7^ ^8^ up to 70% of PID cases have an unidentified aetiology. Bacterial vaginosis-associated organisms have been associated with PID,^9^ ^10^ and because *M genitalium* is associated with cervicitis^4^ ^5^ it is reasonable to hypothesise that it also causes PID. Indeed, this organism induces salpingitis in monkeys,^11^ causes morphological changes in ciliated fallopian tube cells^12^ and has been detected in fallopian tube tissue in a woman with salpingitis.^13^ Whereas studies have associated *M genitalium* with PID and endometritis,^14^^–^16 the efficacy of commonly used PID antimicrobials in treating *M genitalium* upper genital tract infection is unknown.

## MATERIALS AND METHODS

### Patient population

Women who participated in the PID Evaluation and Clinical Health (PEACH) Study and had previously collected and stored cervical and endometrial specimens were studied. The PEACH Study methods have been described in detail elsewhere.^7^ Briefly, women aged 14 to 37 years were recruited between March 1996 and February 1999 from emergency departments, obstetrics and gynaecology clinics, sexually transmitted disease (STD) clinics and private practices at 13 US clinical sites. Altogether, 831 women with clinically suspected PID, defined by pelvic pain of less than 30 days duration, pelvic organ tenderness and leukorrhea, mucopurulent cervicitis or untreated cervicitis were enrolled. For this subsequent *M genitalium* study, stored endometrial and cervical specimens were available for 611 and 682 of participants, respectively.

### Randomisation and treatment

Patients in the parent PEACH study were randomised to inpatient or outpatient antibiotic treatment. The inpatient regimen consisted of cefoxitin (2 g) parenterally every 6 hours and doxycycline (100 mg) orally twice a day for a total of 14 days. The outpatient regimen consisted of a single dose of cefoxitin (2 g) intramuscularly plus probenecid (1 g) orally, then doxycycline (100 mg) to be taken orally twice a day for 14 days. As treatment modality was not associated with reproductive morbidities in the PEACH study,^7^ we do not distinguish between inpatient and outpatient regimens or include them as a covariate in these analyses.

### *M genitalium* PCR

Previously collected cervical and endometrial specimens stored at −70°C were tested for *M genitalium* using the MgPa-IMW PCR assay targeting the MgPa gene^17^ by technicians masked to endometrial histology, treatment failure and reproductive sequelae. This assay has an analytical sensitivity of 15 genomes^17^ and a high clinical sensitivity and specificity relative to  transcription-mediated amplification (TMA)—another *M genitalium* NAAT assay.^18^ For all samples testing positive, a second MgPa PCR assay was performed using another aliquot of the sample to rule out PCR product contamination or cross-contamination; all samples initially positive were verified as positive in this confirmatory test.

### Endometrial biopsies and microbiological studies

At baseline and 30 days in the parent PEACH study, an endometrial biopsy was obtained for histology, chlamydial PCR (Roche Diagnostics, USA), and gonococcal culture. In a subset of 352 women (240 women in the current sub study), endometrial specimens were cultured for *Mycoplasma hominis* and *Ureaplasma urealyticum*.^19^ Endometrial histology was determined based on haematoxylin and eosin stained and methyl green pyronine stained biopsy tissue specimens, read separately by two reference pathologists who reviewed the slide together to reach consensus upon disagreement. A modification^20^ of the criteria proposed by Kiviat *et al*^8^ was used to define endometritis. A classification of endometritis was given upon finding at least five neutrophils in the endometrial surface epithelium in the absence of menstrual endometrium and/or at least two plasma cells in the endometrial stroma. Cervical swabs were used for *N gonorrhoeae* cultures and *C trachomatis* PCR assays. Vaginal smears were Gram stained for bacterial vaginosis as described by Nugent *et al*.^10^

### Follow up

Participants were monitored with in-person visits at 5 and 30 days. Subsequent telephone interviews were conducted every 3 months during the first year and then every 4 months until June 2004, at which point we had follow up information for 541 women, representing a mean follow up of 84 months. Reproductive outcomes included the occurrence of a first pregnancy, first live birth, infertility, chronic pelvic pain and recurrent PID. Infertility was defined when a sexually active woman with at least 12 months of follow up did not report conception (positive urine or blood test or doctor’s diagnosis of pregnancy) despite rare or no use of a contraceptive method. Chronic pelvic pain was defined as pain reported during at least two consecutive follow up interviews; thereby, suggesting a minimum of 6 months duration. Recurrent PID was self reported and verified whenever medical records were available (in 45% of the cohort). Recurrent PID was confirmed in 76% of medical records and rates by self report and medical record review were similar.^7^ As few women experienced an ectopic pregnancy during follow up (n = 6), we did not include this as an outcome.

### Statistical methods

Baseline descriptive characteristics were compared between women with and without *M genitalium* using χ^2^ tests of proportions. The cross-sectional association between *M genitalium* and endometritis at baseline was additionally determined using a logistic model, fit with *M genitalium*, age, race, *N gonorrhoeae* and *C trachomatis* as explanatory variables. Relative risks were calculated for the prospectively determined outcomes. Incident endometritis was defined by a negative histological test for endometritis at baseline followed by a positive histological categorisation for endometritis 30 days following treatment. To examine treatment failure, we categorised persistent endometritis as histological endometritis identified at both the baseline and 30 day examinations. Log-binomial regressions determined the relative risks and 95% confidence intervals (CIs) for endometritis at 30 days, incident endometritis, persistent endometritis, recurrent PID, infertility, chronic pelvic pain, pregnancy and live birth, and included *M genitalium*, age and race as explanatory variables. Infertility, pregnancy and live birth models were additionally adjusted for self reported infertility at baseline, and models predicting treatment failure were additionally adjusted for intercourse between baseline and 30 days and self reported partner treatment. Analyses were conducted first using both cervical and endometrial *M genitalium* PCR results combined and were then repeated using only endometrial PCR. All analyses were conducted in the entire cohort and in a subset of women with negative tests for *N gonorrhoeae* or *C trachomatis*. SAS version 9.1 for Windows was used for all statistical comparisons.

## RESULTS

We detected *M genitalium* in 88 (15%) of women at baseline. Altogether, 11% of cervical specimens and 8% of endometrial specimens were positive for *M genitalium*; infections at these sites were highly correlated (Phi correlation 0.63, p<0.0001), with 74% of endometrial positive cases also positive at the cervix and 60% of cervical positive cases also positive in the endometrium.

Women who tested positive compared to those who tested negative for *M genitalium* were more than twice as likely to have histologically confirmed endometritis at baseline (odds ratio (OR) 2.6, 95% CI 1.5 to 4.6; table 1). After adjustment for age, race, *N gonorrhoeae* and *C trachomatis*, women testing positive for *M genitalium* were two times more likely to have baseline endometritis (OR 2.0, 95% CI 1.0 to 4.2). Participants who tested positive and negative for *M genitalium* had similar scores for self rated pelvic pain at baseline. However, whereas women who tested positive for *M genitalium* alone scored their pelvic pain similarly to those who tested positive for *C trachomatis* alone (p = 0.5), women testing positive for *M genitalium* alone had self rated pelvic pain scores, which were significantly lower than those among women testing positive for *N gonorrhoeae* alone at baseline (58.0 (SD 21.9) vs 72.3 (SD 23.9), p = 0.01). Women with versus those without *M genitalium* identified in endometrial and/or cervical specimens were more likely to be less than 25 years of age, have concurrent *N gonorrhoeae* and *C trachomatis*, and report vaginal douching, drug use and cigarette smoking (table 1).

**Table 1:** Characteristics of women with and without *Mycoplasma genitalium* identified in the endometrium and/or cervix

Characteristic	Mg+ n = 88, n (%)	Mg− n = 498, n (%)	p Value
Histological endometritis*	43/63 (68.3)	197/439 (44.9)	0.0005
Pelvic pain score† (mean SD)	67.7 (SD 21.3)	65.6 (SD 21.5)	0.392
Co-infection			
*N gonorrhoeae* and/or *C trachomatis*	43/65 (66.2)	172/435 (39.5)	< 0.0001
*N gonorrhoeae* alone	26/59 (44.1)	111/423 (26.2)	0.0045
*C trachomatis* alone	23/74 (31.1)	91/484 (18.8)	0.0147
*M hominis*	4/37 (10.8)	17/203 (8.4)	0.6295
*U urealyticum*	1/37 (2.7)	15/203 (7.4)	0.2932
BV	51/80 (63.8)	254/453 (56.1)	0.2006
Demographic			
Age			
< 25 years	71/88 (80.7)	319/498 (64.1)	
⩾ 25 years	17/88 (19.3)	179/498 (35.9)	0.0023
Race/ethnicity			
African American	69/88 (78.4)	358/498 (71.9)	
White/Hispanic/Other	19/88 (21.6)	140/498 (28.1)	0.2047
Marital status			
Unmarried	71/78 (91.0)	411/466 (88.2)	
Married	7/78 (9.0)	55/466 (11.8)	0.4669
Education			
< High school	38/88 (43.2)	189/497 (38.0)	
⩾ High school	50/88 (56.8)	308/497 (62.0)	0.3605
Sexual history			
History of STD‡	51/86 (59.3)	288/485 (59.4)	0.9890
History of BV	13/86 (15.1)	117/482 (24.3)	0.0626
Sexually active	78/88 (88.6)	415/498 (83.3)	0.2095
Rare/occasional condom use§	60/78 (76.9)	289/415 (69.6)	0.1943
Consistent condom use¶	7/78 (9.0)	57/415 (13.7)	0.2511
⩾ 2 sexual partners	10/88 (11.4)	46/498 (9.2)	0.5316
Behavioural			
Vaginal douche, 2+ times in past month	26/88 (29.6)	88/498 (17.7)	0.0095
Drug use	32/88 (36.4)	125/495 (25.3)	0.0304
Current smoker	49/88 (55.7)	194/495 (39.2)	0.0038

*⩾5 surface epithelium neutrophils per ×400 field absent of menstrual endometrium and/or ⩾2 stromal plasma cells per ×120 field; †mean composite pain score  =  (mean of current pelvic pain score, average pelvic pain score and worst pelvic pain score) × 10; ‡history of *Neisseria gonorrhoeae*, *Chlamydia trachomatis* or *Trichonomas vaginalis*; §condoms used 0 to 5 out of 10 sexual encounters; ¶condoms used in 10 out of 10 sexual encounters.

BV, bacterial vaginosis; STD, sexually transmitted disease.

Overall, 23 out of 56 women (41%) with *M genitalium* identified in either the cervix and/or endometrium at baseline had *M genitalium* persistently identified 30 days following treatment. Of women with endometrial specimens obtained at both baseline and 30 days, 44% (14/32) had persistent endometrial *M genitalium* detected. Women with *M genitalium* identified in the endometrium were 4.5 times more likely to experience short-term treatment failure, defined by histological identification of endometritis and persistent pelvic pain at the 30 day follow up clinic visit (adjusted rate ratio (RR) 4.6, 95% CI 1.1 to 20.1; table 2). Having *M genitalium* identified in the endometrium at baseline was associated with both an increased risk of incident endometritis at 30 days (adjusted RR 5.0, 95% CI 1.2 to 20.6) and persistent endometritis, identified histologically at both baseline and 30 days following treatment (adjusted RR 4.1, 95% CI 1.4 to 11.9). In models additionally adjusted for *N gonorrhoeae* and *C trachomatis*, endometrial *M genitalium* was associated with incident endometritis (adjusted RR 6.0, 95% CI 1.4 to 27.1) and treatment failure (adjusted RR 5.2, 95% CI 0.9 to 30.6), although the association with treatment failure was of borderline statistical significance. Results were similar among a subset of women testing negative for *C trachomatis* and *N gonorrhoeae*, although cell sizes were small and confidence intervals large. In this group of women without *N gonorrhoeae* or *C trachomatis*, those with *M genitalium* identified in the endometrium at baseline were nearly nine times as likely to be categorised as having endometritis at the 30 day examination (adjusted RR 8.8, 95% CI 2.0 to 39.6) and over 13 times as likely to be defined as having incident endometritis at 30 days (adjusted RR 13.4, 2.4 to 75.2). As co-infection was common in this study, models with microbiological interaction terms were additionally examined. *M genitalium* did not significantly interact with *N gonorrhoeae* or *C trachomatis* to additionally influence the risk of incident endometritis or treatment failure. In models adjusting for *M hominis* and *U urealyticum*, *M genitalium* was associated with almost a 2.5-fold increased risk of persistent endometritis (RR 2.4, 95% CI 0.5 to 12.4). However, as *M hominis* and *U urealyticum* were only cultured on a subset of women in the parent PEACH study, a smaller sample size resulted in a wide confidence interval.

**Table 2:** Endometritis 30 days following treatment with cefoxitin and doxycycline and short-term treatment failure (endometritis at 30 days and persistent pelvic pain) among women with and without baseline *Mycoplasma genitalium* (MG) genital tract infection

Outcome	Cervical and/or endometrial Mg+ n (%) n = 88		Cervical and/or endometrial Mg− n (%) n = 498	Adjusted RR¶ (95% CI)	Endometrial Mg+ n (%) n = 50	Endometrial Mg− n (%) n = 561	Adjusted RR¶ (95% CI)
Treatment failure*	8 (40.0)	26 (17.5)		2.6 (0.8 to 8.9)	6 (50.0)	30 (18.5)	4.6 (1.1 to 20.1)
Endometritis at 30 days†	25 (56.8)	119 (39.0)		1.9 (0.9 to 3.9)	19 (70.4)	131 (39.2)	4.0 (1.5 to 10.9)
Incident endometritis‡	6 (24.0)	35 (15.8)		1.5 (0.4 to 5.0)	5 (38.5)	37 (15.4)	5.0 (1.2 to 20.6)
Persistent endometritis§	17 (47.2)	73 (28.2)		2.1 (1.0 to 4.6)	13 (61.9)	82 (28.8)	4.1 (1.4 to 11.9)

*Treatment failure is defined as both endometritis and pelvic pain present at the 30 day clinic visit; †All women categorised as having endometritis at the 30 day clinic visit; ‡includes women without endometritis at baseline who are categorised as having endometritis at the 30 day clinic visit; §includes women who are categorised as having endometritis at both baseline and 30 days; ¶Adjusted for age and race, self reported partner treatment and intercourse between baseline and the 30 day clinic visit.

Rates of infertility (22%), recurrent PID (31%) and chronic pelvic pain (42%) were high among women testing positive for endometrial *M genitalium* at baseline. Although the rates of reproductive morbidity were not significantly higher among women testing positive for *M genitalium*, all relationships were in the hypothesised direction (table 3). Women with versus those without endometrial *M genitalium* experienced modest, non-significantly elevated risks of recurrent PID, infertility and chronic pelvic pain, and were slightly but non-significantly less likely to experience a pregnancy or live birth.

**Table 3:** Sequelae and long-term treatment failure among women with and without baseline *Mycoplasma gentalium* (MG) genital tract infection

Outcome	Cervical and/or endometrial Mg+ n (%) n = 88	Cervical and/or endometrial Mg− n (%) n = 498	Adjusted RR* (95% CI)	Endometrial Mg+ n (%) n = 50	Endometrial Mg− n (%) n = 561	Adjusted RR* (95% CI)
Recurrent pelvic inflammatory disease	24 (28.2)	98 (20.8)	1.6 (0.8 to 3.1)	15 (30.6)	111 (20.8)	1.8 (0.8, 3.8)
Infertility	15 (17.4)	95 (19.4)	1.4 (0.6 to 2.9)	11 (22.0)	104 (18.9)	1.1 (0.7 to 4.0)
Chronic pelvic pain	37 (43.5)	198 (41.4)	1.6 (0.9 to 2.9)	21 (42.0)	226 (42.1)	1.3 (0.6 to 2.7)
Pregnancy	53 (61.6)	278 (56.9)	0.8 (0.4 to 1.5)	29 (58.0)	317 (57.6)	0.7 (0.3 to 1.4)
Live birth	34 (50.7)	207 (48.5)	0.7 (0.3 to 1.3)	19 (46.3)	236 (49.3)	0.6 (0.3 to 1.3)

*All models are adjusted for age, race, *Neisseria gonorrhoeae* and *Chlamydia trachomatis*. Infertility, pregnancy and live birth models are additionally adjusted for self reported infertility at baseline.

## DISCUSSION

We detected *M genitalium* at a high prevalence (15%) among an urban US population of women with non-gonococcal, non-chlamydial PID, similar to that found in prior PID studies among Kenyan (16%)^16^ and UK women (13%).^15^ In the PEACH study, about 14% of women were infected with *C trachomatis*, 15% were infected with *N gonorrhoeae* and 5% were co-infected with these two pathogens.^7^ Thus, *M genitalium* was as prevalent as *C trachomatis* or *N gonorrhoeae* among this population of women with PID.

Women with endometrial *M genitalium* were over three times more likely to have histologically confirmed endometritis compared to women without *M genitalium* identified at this site. Our study suggests that the relationship between *M genitalium* and endometritis is independent and causal, since among women without concurrent *N gonorrhoeae* and/or *C trachomatis*, endometrial *M genitalium* was associated with over a 13-fold risk of incident endometritis at the 30 day follow up visit. Our results are consistent with previous cross-sectional studies associating *M genitalium* with PID.^14^ ^16^ Further, we found that although pelvic pain was similar between women testing positive and negative for *M genitalium*, women who tested positive for *M genitalium* alone reported significantly less pelvic pain than women who tested positive for *N gonorrhoeae* alone. Pelvic pain scores were lower and similar among women who tested positive for *M genitalium* alone and *C trachomatis* alone, suggesting that, like *C trachomatis*, *M genitalium* may also play a role in asymptomatic or “silent” PID.

To our knowledge, ours is the first study to investigate treatment failure among PID patients with *M genitalium* identified in the endometrium. Persistence of *M genitalium* following treatment with a standard Centers for Disease Control and Prevention recommended PID treatment regimen was very high: 44% of women with baseline endometrial positive specimens tested positive again 30 days following treatment. In contrast, only 2–4% of women in the PEACH study had persistent or recurrent gonococcal or chlamydial cervicitis when retested at 30 days.^7^ Women with *M genitalium* identified in the endometrium at baseline were four times as likely to experience persistent endometritis and approximately 4.5 times as likely to experience treatment failure—defined as the presence of both endometritis and pelvic pain 30 days following treatment for PID.

Several attributes of *M genitalium* indicate that it is resistant to cefoxitin and doxycycline. First, mycoplasmal bacteria lack a cell wall and are thus resistant to cell wall inhibiting antibiotics. Second, an *M genitalium* strain with increased tetracycline resistance has been isolated^21^ and *M genitalium* is associated with persistent non-gonococcal urethritis among men treated with tetracyclines.^22^ Most women with PID are treated with antibiotics directed toward *N gonorrhoeae* and/or *C trachomatis*, despite the fact that these pathogens account for only a third to a half of PID cases. Indeed, in the PEACH study approximately 60% of women had non-gonococcal, non-chlamydial PID.^7^ Over a third of the women had persistent endometritis post treatment,^7^ identifying a sizeable portion as having ongoing upper genital tract inflammation and demonstrating that antibiotics used to treat this critically important upper tract syndrome are suboptimal. *M genitalium* has demonstrated variable resistance to fluoroquinolones^23^ and susceptibility to macrolides, although azithromycin resistant strains have recently been identified.^24^ A newer quinolone, moxifloxacin, has recently been shown to exhibit a high degree of activity against *M genitalium*.^21^ Randomised clinical trials assessing these alternative regimens for the treatment of *M genitalium* and PID are needed.

It is possible that other organisms resistant to doxycycline or cefoxitin may have confounded the relationship between *M genitalium* and persistent endometritis. However, *M genitalium* was not associated with endometrial *M hominis* or *U urealyticum*—organisms that express tetracycline resistance^25^ and contain *tet*M genes that encode tetracycline resistance.^26^ Further, adjustment for these pathogens only slightly attenuated the relationship between *M genitalium* and persistent endometritis. Additionally, we recognise that PID may recur among women who engage in risky sexual behaviour during treatment, and this may also confound treatment failure comparisons. However, in models adjusted for self reported partner treatment and intercourse, *M genitalium* was significantly predictive of treatment failure, supporting the idea that *M genitalium* antibiotic resistence may lead to persistent PID.

Although a positive *M genitalium* test was not significantly predictive of reproductive morbidity in our study, results were in the hypothesised direction. Infertility, chronic pelvic pain and recurrent PID were more common among women testing positive for endometrial *M genitalium*, and women who tested positive were less likely to become pregnant or have a live birth. Certainly, these reproductive outcomes are more subject to misclassification and bias than shorter term outcomes due to loss of follow up and reliance on self reported data. Such misclassification may have biased our results towards the null. Additionally, it is possible that women may have had prior *M genitalium* infection that resulted in tubal damage preceding the baseline PEACH study PID episode. As such prior infection would not be identified using PCR, which identifies acute infection, this may have biased our results towards the null. Indeed, in a study of 308 women undergoing in vitro fertilisation treatment, women determined to have tubal factor infertility were over three times as likely to be seropositive for *M genitalium*.^27^ Moreover, among a study of 212 couples attending infertility clinics, although *M genitalium* was not detected by PCR in any cervical swab specimens, *M genitalium* seropositivity was strongly associated with tubal factor infertility.^28^ This supports the idea that previous *M genitalium* infection may result in permanent damage and occlusion of fallopian tubes. Lastly, although we found *M genitalium* to be associated with treatment failure, we have previously reported that short-term markers, including pelvic tenderness at 5 and 30 days, are not predictive of reproductive morbidity in the PEACH study population.^29^ As over two-thirds of women in the PEACH study presented for treatment with 3 or more days of pelvic pain,^7^ and since delayed care for PID is associated with almost a threefold increased risk for infertility,^30^ all women in the PEACH study may have been at increased risk for reproductive sequelae regardless of short-term treatment response. Indeed, women in the PEACH study experienced distressingly high rates of morbidity regardless of microbial aetiology of PID (infertility 17%,^7^ recurrent PID 14%^7^ and chronic pelvic pain 37%^31^). Certainly, women with *M genitalium* identified in either the cervix or endometrium had high rates of infertility (18%), recurrent PID (28%) and chronic pelvic pain (44%). These infertility rates are approximately 2.5 times higher than that reported from a study utilising 2002 National Survey of Family Growth data,^32^ suggesting that preservation of fertility may be suboptimal for many women treated for PID.

Recommended PID treatment regimens are based on clinical trial data demonstrating overall high rates of clinical cure. PID is well recognised though as having a polymicrobial aetiology, and not all microbial infections may respond equally well to the same antimicrobial. In our study of women with clinically suspected PID, we found *M genitalium* to be associated with endometritis and short-term PID treatment failure, as evidenced by persistent endometritis and continued pelvic pain. We conclude that cefoxitin and doxycycline, a Center for Disease Control recommended PID treatment regimen, is ineffective for the treatment of *M genitalium* upper genital tract infection.

**Key messages**

- *Mycoplasma genitalium* is frequently detected from the cervix and endometrium of women with clinically suspected pelvic inflammatory disease (PID).
- *M genitalium* is associated with endometritis and short-term PID treatment failure, as evidenced by persistent endometritis and continued pelvic pain.
- Cefoxitin and doxycycline, a Center for Disease Control recommended PID treatment regimen, is ineffective for the treatment of *M genitalium* upper genital tract infection.
